# Effects of ultrafiltration on Co-Metal Organic Framework/pre-hydrolysis solution carbon materials for supercapacitor energy storage

**DOI:** 10.3389/fchem.2022.991230

**Published:** 2022-08-16

**Authors:** Changwei Li, Lei Sha, Kang Yang, Fangong Kong, Peng Li, Yubo Tao, Xin Zhao, Honglei Chen

**Affiliations:** State Key Laboratory of Biobased Material and Green Papermaking, Qilu University of Technology, Shandong Academy of Sciences, Jinan, China

**Keywords:** Co-MOF, pre‐hydrolysis, carbon, hydrothermal carbonization, supercapacitor

## Abstract

Here, a Co-Metal Organic Framework/pre-hydrolysis (Co-MOF/pre-hydrolysis) solution carbon material is prepared by a mild and environmentally-friendly hydrothermal carbonization technique using a pulping pre-hydrolysis solution as the raw material and Co-MOF as the metal dopant. The stable hollow structure provide sufficient space for particle shrinkage and expansion, while the low density and large specific surface area of the long, hairy tentacle structure provide a greater contact area for ions, which shorten the transmission path of electrons and charges. The materials exhibit excellent specific capacitance (400 F/g, 0.5 A/g) and stability (90%, 10,000 cycles). The Change of different concentration ratios in the structures significantly affect the electrochemical performance. The specific surface area of the carbon materials prepared by ultra-filtration increased, but the specific surface area decrease as ultrafiltration concentration increase. The specific capacitance decrease from 336 F/g for C-ZIF-67-1/3 volume ultrafiltration to 258 F/g for C-ZIF-67-1/5 ultrafiltration. The results indicate that energy storage by the carbon materials relied on a synergistic effect between their microporous and mesoporous structures. The micropores provide storage space for the transmission of ions, while the mesopores provide ion transport channels. The separation of large and small molecules after ultrafiltration concentration limit the ion transmission and energy storage of the pores.

## Introduction

Due to increasingly serious environmental problems, clean energy has obtained unprecedented development opportunities, but energy storage and conversion are technical bottlenecks that restrict the development of new energy industries; thus, supercapacitors have received extensive attention as energy storage and conversion devices (Li et al., 2021a). Carbon materials are ideal materials for double-layer capacitors due to their abundant reserves, low cost, high specific surface area, and tunable pore structures (Du et al., 2021; Zhang et al., 2021). The power density of carbon-based supercapacitors is mainly influenced by the degree of graphitization of carbon materials-the higher the degree of graphitization, the better the conductivity (Saini et al., 2021), however, the degree of graphitization of bio-based carbon materials is relatively low due to their complex structures and uncontrollable composition (Zhou et al., 2021a). To optimize the electrochemical performance of bio-based carbon materials, high-energy storage metal/bio-based carbon materials can be prepared by adding small amounts of metal materials to regulate the morphology of carbon materials while providing capacitance support (Jia et al., 2021).

In the pulp and paper industry, high-purity refined pulp produces pre-hydrolysis solution as a by-product, which is mainly composed of lignin, hemicellulose, and degradation products. It can be used to prepare biomass-based carbon materials (Wu et al., 2021). Meanwhile, liquid-phase biomass can provide favorable homogeneous conditions for reactions to efficiently produce stable composites. If this by-product can be fully utilized, high value-added resource utilization of waste can be achieved; however, whether the homogenization of molecules in the pre-hydrolysis solution will improve the electrochemical performance of carbon materials remains unclear. As a molecular separation technique, ultra-filtration enables small molecules in the original liquid to pass through the membrane from the high-pressure side to the low-pressure side (Liu et al., 2018). In this way, small and large molecules in the pre-hydrolysis solution can be separated to homogenize the small carbohydrates in the pre-hydrolysis solution (Rahmaninezhad et al., 2020).

In this study, ultra-filtration was used to separate the large and small molecules in a pre-hydrolysis solution and homogenize small carbohydrate molecules. Using the ultra-filtrated pre-hydrolysis solution as the carbon source and Co-MOF as the metal dopant, Co-MOF/pre-hydrolysis solution carbon materials were prepared by a hydrothermal process. The effect of the morphology and pore structure of the prepared composites on the electrochemical performance of the obtained materials was investigated to determine how the structures of macromolecules and how small molecules affected the electrochemical performance of the carbon materials for energy storage applications.

## Experimental

### Preparation of pre-hydrolysis solution

Poplar wood and deionized (DI) water were mixed in a vessel at a mass ratio of 1:7 and heated at 175°C for 2 h. The resulting mixture was filtered by polymerized maple ultrafiltration membrane (3k) and centrifuged at 8,000 r/min to obtain a homogeneous pre-hydrolysis solution. The volume ratio of the solution is set to be 1.

### Preparation of ultra-filtrated pre-hydrolysis solutions

The pre-hydrolysis solution of volume ratio 1 was added to an ultrafiltration cup. The ultrafiltration membrane was a 3 kd polyethersulfone membrane, and the ultrafiltration was concentrated to 1/3 volume, 1/4 volume, and 1/5 volume, respectively.

### Preparation of Co-MOF

Cobaltous nitrate hexahydrate (2.328 g) and dimethylimidazole (2.627 g) were dissolved in 100 ml of anhydrous methanol. Subsequently, the two solutions were mixed and stirred for 30 s. The reaction was continued at room temperature for 24 h without stirring. After the reaction, a purple solid was collected by centrifugation and precipitation and then washed repeatedly with anhydrous methanol and dried overnight at 80°C under vacuum to obtain Co-MOF (ZIF-67) purple powder.

### Preparation of Co-MOF/carbon materials

The prepared ZIF-67 (1 g) and ultra-filtrated pre-hydrolysis solution (ultrafiltrated and concentrated to 1, 1/3, 1/4, 1/5) (100 ml) were mixed and stirred for 30 s. The mixtures were then heated in a PTFE reactor at 250°C for 12 h and then cooled to room temperature. The solids were separated by centrifugation and precipitation, washed repeatedly with DI water, and dried at 80°C. The black solid was carbonized at 800°C for 2 h under a protective N_2_ atmosphere at a heating rate of 5°C/min. Samples were recorded as C-ZIF-67, C-ZIF-67-1/3, C-ZIF-67-1/4, and C-ZIF-67-1/5, respectively.

### Characterization

Samples were characterized by a field emission scanning electron microscope (20 kV, FESEM, JSM-7401F, JEOL, Hokkaido, Japan) and transmission electron microscope (200 kV, TEM, JEOL 2011, JEOL, Japan). X-ray powder diffraction (XRD) patterns of samples were obtained using a Bruker D4 (Bruker, Rheinstetten, Germany) X-ray powder diffractometer at 40 kV and 40 mA. Raman spectra were recorded using a laser confocal Raman spectrometer (inVia; Raman, Renishaw, Gloucestershire, England) with a laser wavelength of 532 nm. Nitrogen adsorption isotherms were measured at 77 K using a Micromeritics ASAP 2020 gas adsorption apparatus (Maike, Atlanta, GA, United States) with nitrogen as the adsorbent. The pore size distribution (PSD) data were obtained by the Barrett-Joyner-Halenda (BJH) method using a slit pore model.

### Electrochemical testing

The electrochemical performance of ZIF-67/carbon materials was measured by a three-electrode system in a 1 M KOH aqueous electrolyte. The obtained carbon materials, carbon black, and polytetrafluoroethylene (PTFE) with a mass ratio of 8:1:1 were used to prepare the working electrode. The mixture (6 mg) was coated on a 1.5 cm × 1.5 cm nickel foam and dried at 60°C for 8 h before pelleting. Cyclic voltammetry (CV) curves and galvanostatic charge/discharge (GCD) curves were measured on a PARSTAT 4000A electrochemical workstation. The specific capacitance of the electrode was calculated from the galvanostatic charge/discharge curves according to the following equation (Wang et al., 2019):

C=IΔt/(mΔV) (1)

where *C* is the specific capacitance (F/g), *I* is the current (A), *m* is the mass of the active material in the electrode (g), *ΔV* is the potential difference (V) in *Δt* (s) at a given discharge.

The energy density, *E* (Wh/kg), and power density, *P* (W/kg ), of each sample were calculated from the discharge plots using Eqs 2, 3, respectively:

$$E=\left(C\times \Delta V^{2}\right)/2$$ (2)

P=E/Δt (3)

## Results and discussion

**FIGURE 1:**
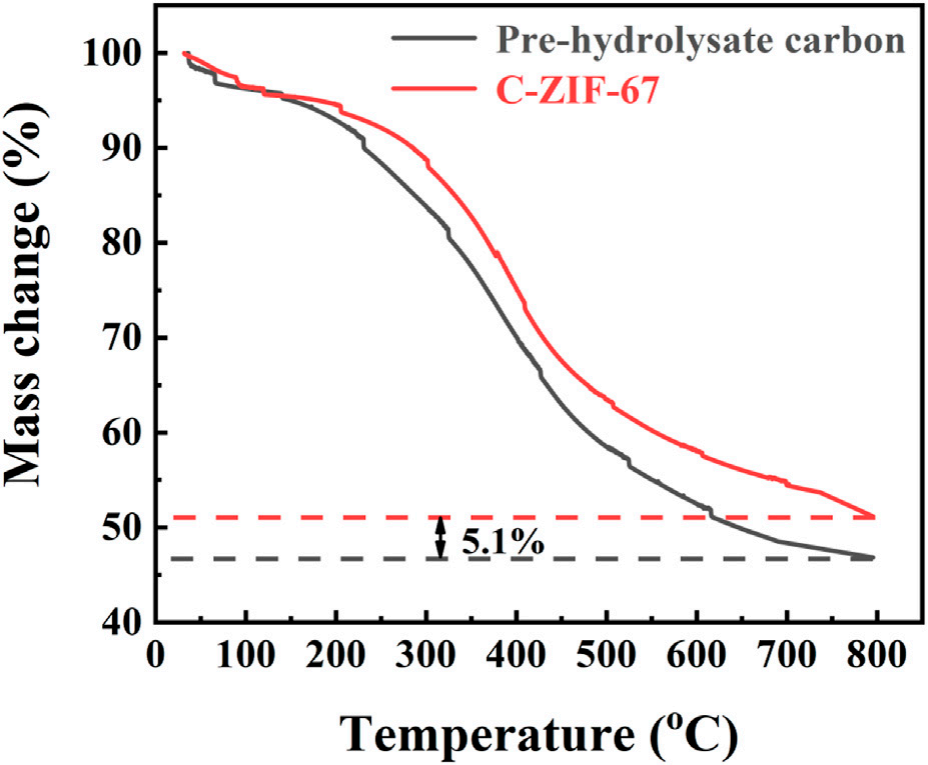
Thermogravimetry images of the pre-hydrolysate and C-ZIF-67 precursors.

The thermogravimetric images of the pre-hydrolysate and C-ZIF-67 precursors are shown in Figure 1. The decomposition of the samples could be divided into three main stages. The weight loss was approximately 5 wt% at the first stage (0°C–150°C), which was attributed to the volatilization of water (combined water and free water) (Poletto et al., 2014). The weight loss is approximately 42 wt% and 47% at the second stage (150°C–700°C) of the pre-hydrolysate and C-ZIF-67 precursors which was mainly the decomposition of lignin and other macromolecules, and the reason for the lower weight loss of C-ZIF-67 is that the stability property of Co-MOF at this temperature range (Collard and Blin, 2014). After the degradation of the macromolecules, the weight loss rate was slightly decreased (about 4 wt% and 2% of the C-ZIF-67 and pre-hydrolysate carbon precursor), which resulted from the small molecule volatilization of CO_2_, CH_4_, and CO during the third stage (700°C–800°C) (Vilella et al., 2017). At this stage, C-ZIF-67 lost slightly more weight than pre-hydrolysate carbon, probably because Co-MOF decomposed more at high temperature. In addition, the final weight of the Co-MOF and pre-hydrolysate carbon are 51.15% and 46.05%, so the metal loading is 5.1% in material. The low loading of metals enough to improve electrochemistry performance of the MOF/pre-hydrolysate carbon in this work.

**FIGURE 2:**
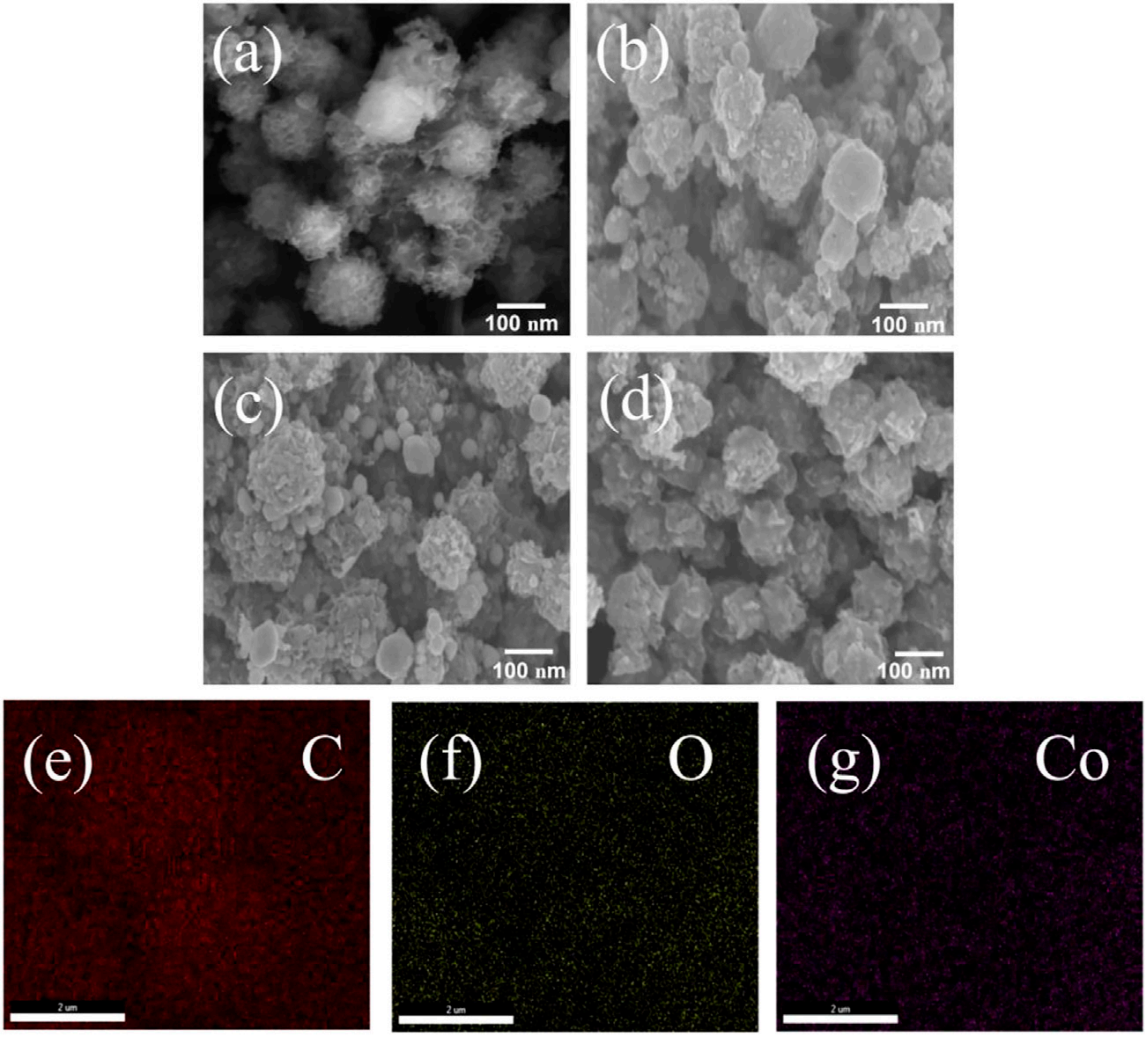
SEM images of **(A)** C-ZIF-67, **(B)** C-ZIF-67-1/3, **(C)** C-ZIF-67-1/4, and **(D)** C-ZIF-67-1/5, EDS of **(E)** C elemental, **(F)** O elemental, and **(G)** Co elemental mapping for C-ZIF-67.

Figures 2A–D shows SEM images of different samples. The particle size of range C-ZIF-67 was 0.35–0.90 μm, and it had a rough three-dimensional flower-like structure (Figure 2A) with fluffy tentacles on its surface. This special morphology increased the contact area between carbon materials and the electrolyte, which shortened the transmission path of electrons and improved the capacitance of carbon materials (Zheng et al., 2021). After concentration, the morphology of the material was changed. C-ZIF-67-1/3 showed rough irregular spheres (Figure 2B) with small raised particles on the sphere surfaces, which provided more active sites and increased the effective contact area, which is beneficial to supercapacitors. The particle size range of C-ZIF-67-1/3 was 150–300 nm, possibly due to the inhomogeneous distribution of different types of sugars (e.g., ribose, xylose, and arabinose) in the pre-hydrolysis solution. C-ZIF-67-1/4 (Figure 2C) and C-ZIF-67-1/5 (Figure 2D) also exhibited rough irregular spherical structures with small raised particles on their surfaces, but most C-ZIF-67-1/4 and C-ZIF-67-1/5 particles had a size of 100 nm. The particle size distributions of C-ZIF-67-1/4 and C-ZIF-67-1/5 were significantly narrower than that of C-ZIF-67-1/3, probably because the degree of concentration continued to increase (Annamalai et al., 2022). The small sugar molecules (e.g., ribose, xylose, and arabinose) in the pre-hydrolysis solution tended to homogenize, and the size of carbon spheres became more uniform (Gao et al., 2021). The changes in size and structure inevitably affected the electrochemical performances of the metal/carbon composites. In Energy dispersive spectroscopy (EDS) image (Figures 2E–G), C-ZIF-67 is dominated by carbon, uniformly decorated with oxygen and cobalt, which have a positive effect on pseudo-capacitance from effective oxygen-containing functional groups and Co-MOF.

**FIGURE 3:**
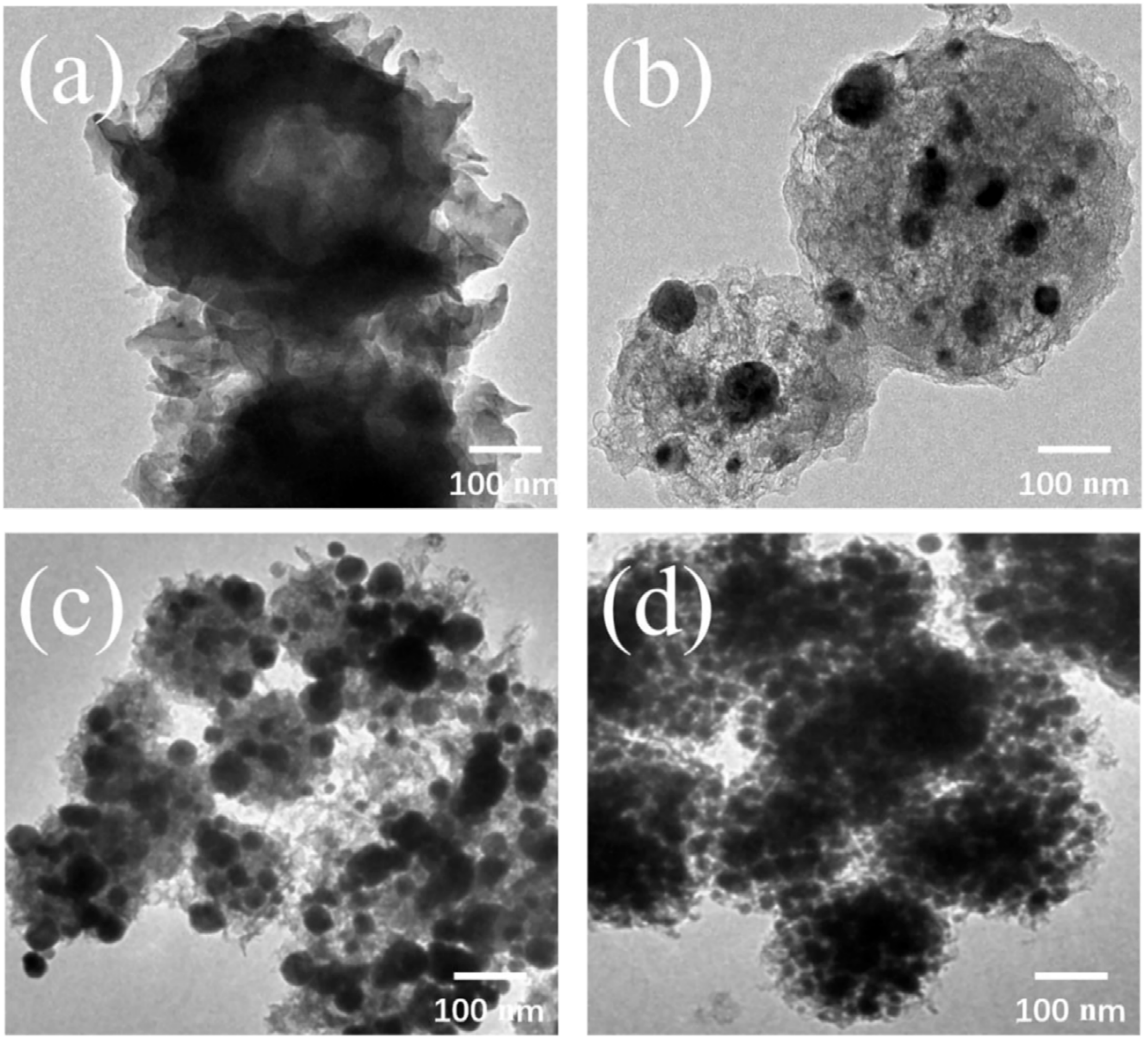
TEM images of **(A)** C-ZIF-67, **(B)** C-ZIF-67-1/3, **(C)** C-ZIF-67-1/4, and **(D)** C-ZIF-67-1/5.

The TEM images of the samples are shown in Figure 3. C-ZIF-67 showed a 150 nm fluffy structure with a hollow interior (Figure 3A), which was probably due to carbonization of the carbon source in the liquid on the Co-MOF surface during the hydrothermal process and encapsulation, followed by the decomposition of the Co-MOF interior during high-temperature carbonization. The hollow structure had a low density and large specific surface area, which provided more active sites and a greater electrolyte electrode contact area (Eftekhari, 2019). It also provided enough space for the shrinkage and expansion of particles (Dahal et al., 2019).

C-ZIF-67-1/3 showed a large spherical structure with a size range of 150–300 nm, with irregular 50 nm spherical particles attached to its surface. C-ZIF-67-1/4 showed a spherical structure with a size of 100 nm, with 30–50 nm irregular spherical particles attached to its surface. C-ZIF-67-1/5 showed a spherical structure with a size of 100 nm, with spherical particles (30 nm) attached to its surface. This was consistent with the SEM images. These results indicate that the concentration affected the morphology of the carbon materials and that the size of carbon spheres in the pre-hydrolysis solution was uniform. This was because concentration via ultrafiltration separated the small and large molecules in the pre-hydrolysis solution, so that small sugar molecules in the pre-hydrolysis solution became homogenized. This homogenization might affect the energy storage properties of the material.

**FIGURE 4:**
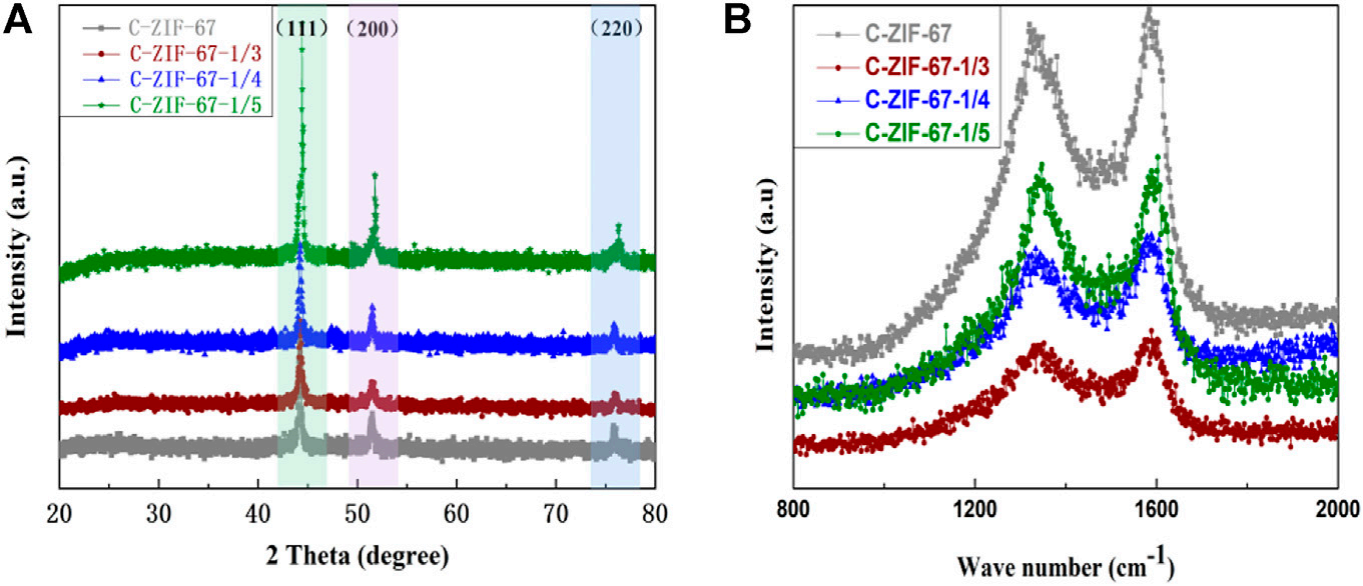
**(A)** XRD patterns and **(B)** Raman spectras of C-ZIF-67, C-ZIF-67-1/3, C-ZIF-67-1/4, and C-ZIF-67-1/5

Figure 4A shows the XRD pattern, in which all samples had a characteristic peak of the (002) crystal plane of graphitic carbon at 25°, indicating the low crystallinity and graphitization of the carbon materials, probably due to the porous structure and defects in the sample (Gu et al., 2020). The (111), (200), and (220) crystal plane peaks of metallic Co appeared at 44.3°, 51.7°, and 75.9° (JCPDS No.15-0806). All samples showed the same characteristic peaks, indicating that all samples had the same crystal structure. Figure 4B shows the Raman spectra of the samples, in which the characteristic peak at 1,350 cm^−1^ (D peak) indicated the disordered graphite structure, and the characteristic peak at 1,590 cm^−1^ (G peak) indicated the graphitic carbon structure of the samples (Zhou et al., 2021b). The disorder of carbon materials was evaluated by the *I*_D_/*I*_G_ ratio (Yu et al., 2018). The *I*_D_/*I*_G_ of the C-ZIF-67 sample was 1, and the *I*_D_/*I*_G_ of C-ZIF-67-1/3, C-ZIF-67-1/4, and C-ZIF-67-1/5 was approximated to be 0.94 (the real values are 0.939, 0.941, and 0.941, respectively), indicating that all samples were graphitized disordered carbon materials (Jiang et al., 2022a).

**FIGURE 5:**
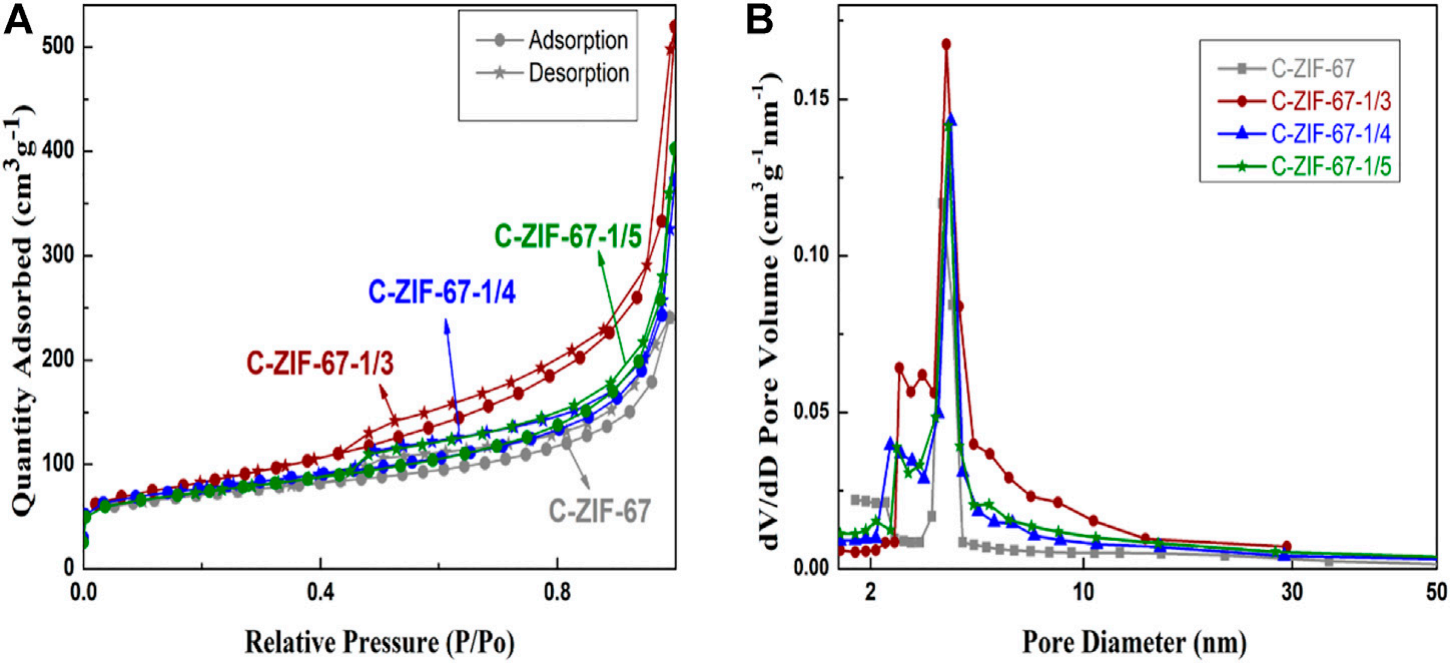
The nitrogen adsorption isotherms **(A)** and the corresponding PSD curves **(B)** of C-ZIF-67, C-ZIF-67-1/3, C-ZIF-67-1/4, and C-ZIF-67-1/5

Figure 5 shows the nitrogen adsorption isotherms (a) and the corresponding PSD curves (b) of the carbon materials. All samples showed typical type IV adsorption curves (Figure 5A), with similar adsorption capacities at lower pressures (*P*/*P*_0_ < 0.02), suggesting that the materials had microporous structures. There were significant hysteresis loops in the relative pressure *P*/*P*_0_ range of 0.45–1.0, which indicated the presence of mesoporous structures (He et al., 2017).

Compared with the C-ZIF-67 sample, the ultra-filtered sample had a more obvious hysteresis loop, indicating that the mesopores content increased after ultrafiltration; however, the mesopores content decreased upon increasing ultrafiltration (Jiang et al., 2022b). Mesoporous structures provide transmission channels for energy storage materials, while micropores provide storage space. This change in the pore structure should affect the energy storage mechanism of the materials (Lin et al., 2018). The pore structures of the samples were in the range of 2.0–5.0 nm (Figure 5B), but the C-ZIF-67 content of samples was higher at the micropores. The pore size of 2.0–5.0 nm could provide suitable paths that facilitate rapid ion transfer (Yu et al., 2017), while the micropores provided space for the adsorption and storage of electrolyte ions (Zhang et al., 2020a). This hierarchical porous structure is very important for supercapacitors.

The pore structure properties of the samples are listed in Table 1. The *S*_BET_ of C-ZIF-67 was 235 m^2^/g, and the total pore volume was 0.252 cm^3^/g, of which *S*_micro_/*S*_BET_ was 85%. After ultrafiltration, the specific surface area and pore volume increased; however, as the concentration ratio increased, the specific surface area decreased from 294 for C-ZIF-67-1/3 to 252 for C-ZIF-67-1/4, and then to 246 m^2^/g for C-ZIF-67-1/5. Increasing the concentration increased the micropore content from 70% in C-ZIF-67-1/3 to 76% in C-ZIF-67-1/4 and then to 80% in C-ZIF-67-1/5, possibly due to the homogenization of small molecules.

**TABLE 1:** Pore structure properties of the samples.

<>Sample	*S*_BET_ (m^2^/g)	*S*_micro_/*S*_BET_ (%)	*V*_total_ (cm^3^/g)
C-ZIF-67	235	85	0.254
C-ZIF-67-1/3	294	70	0.958
C-ZIF-67-1/4	252	76	0.576
C-ZIF-67-1/5	246	80	0.623

**FIGURE 6:**
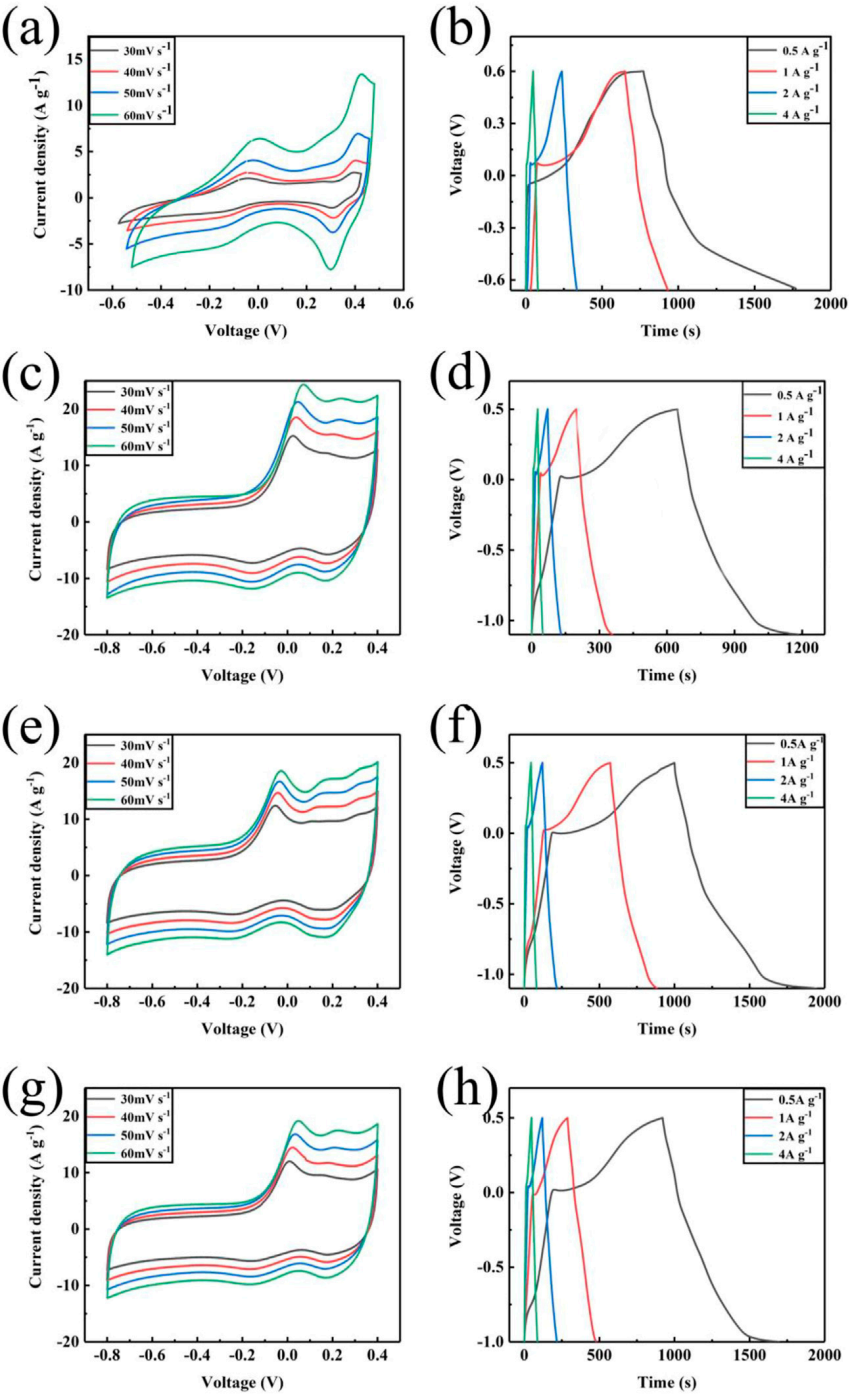
CV curves at different scanning rates: C-ZIF-67 **(A)**, C-ZIF-67-1/3 **(C)**, C-ZIF-67-1/4 **(E)**, and C-ZIF-67-1/5**(G)** and charge/discharge curves at different current densities: C-ZIF-67 **(B)**, C-ZIF-67-1/3 **(D)**, C-ZIF-67-1/4 **(F)**, and C-ZIF-67-1/5 **(H)**.

The electrochemical performances of the prepared samples were measured by CV and GCD tests in a three-electrode system with 1 M KOH aqueous electrolyte (Figure 6). Figure 6A shows the CV curves of C-ZIF-67 at different scanning rates, which displays a rectangular shape and a pair of redox peaks, indicating the double-layer capacitance and pseudo-capacitance of the material (Gu et al., 2020). Upon increasing the scanning rate, the curve shape of C-ZIF-67 did not change significantly because the fluffy structure of C-ZIF-67 increased the contact area between carbon materials and electrolyte ions, which increased the capacitance. The hollow structure did not consume energy generated by internal resistance during the cyclic process (Sui et al., 2022). In addition, the redox peaks of C-ZIF-67 were obvious and symmetric, which further indicated the excellent electrochemical performance of C-ZIF-67 (Li et al., 2020a). The specific capacitance of C-ZIF-67 was 400 F/g at 0.5 A/g (Figure 6B). The CV curves of C-ZIF-67-1/3 (Figure 6C), C-ZIF-67-1/4 (Figure 6E), and C-ZIF-67-1/5 (Figure 5G) at different scanning rates contained redox peaks, which indicated that all samples displayed double-layer capacitance and pseudo-capacitance (Zhang et al., 2020b). Upon increasing the scanning rate, the curve shape of the sample obtained by ultrafiltration did not change significantly, which indicated that the sample structure was stable (Wang et al., 2022). The specific capacitance values of C-ZIF-67-1/3, C-ZIF-67-1/4 and C-ZIF-67-1/5 were 336 F/g (Figure 6D), 294.5 F/g (Figure 6F) and 258 F/g (Figure 6H), respectively, at a current density of 0.5 A/g. These results showed that the specific capacitance of the carbon materials decreased due to ultrafiltration, and the specific capacitance of the materials decreased gradually upon increasing the concentration of the pre-hydrolysis solution by ultrafiltration, which might be due to changes in the pore structure. Carbon materials can be applied in energy storage materials due to synergistic effects between their microporous and mesoporous structures, with micropores providing storage space for ion transmission. The separation of large and small molecules after ultrafiltration concentration will limit the pore transmission and energy storage of ions (Li et al., 2021b).

The energy density E (W h/kg) and power density P (W/kg) of the samples were calculated using Eqs. 2, 3, respectively. The sample of C-ZIF-67 produces a high energy density of 80 W h/kg at a power density of 300 W/kg. And the energy density of C-ZIF-67-1/3, C-ZIF-67-1/4 and C-ZIF-67-1/5 are 67.2, 58.9 and 51.6 W h/kg, respectively, while the power density are 252, 221 and 193.5 W/kg, respectively. Compared with other samples, C-ZIF-67 showed a more excellent electrochemical performance. Actually, many works of MOF/carbon materials have been explored in Table 2, and the obtained sample (C-ZIF-67) in this work has certain advantages in capacitance value.

**TABLE 2:** The specific capacitance of C-ZIF-67 and other materials.

Materials	Electrolyte	Current density (A/g)	Specific capacitance (F/g)	References
Ni_3_(HITP)_2_ EDLC	TEABF_4_/ACN	0.05	111	Sheberla et al. (2017)
PC-Zn	KOH	0.5	138	Yue et al. (2018)
IM-HPC	H_2_SO4	0.5	236	Li et al. (2020b)
Co_3_O_4_@Carbon	KOH	1	261	Dai et al. (2017)
CoAl-LDH@Carbon	H_2_SO_4_	1	300.7	Wu et al. (2020)
CNTs/NCP	H_2_SO4	1	308	Xu et al. (2016)
C-ZIF-67	KOH	0.5	400	This work

**FIGURE 7:**
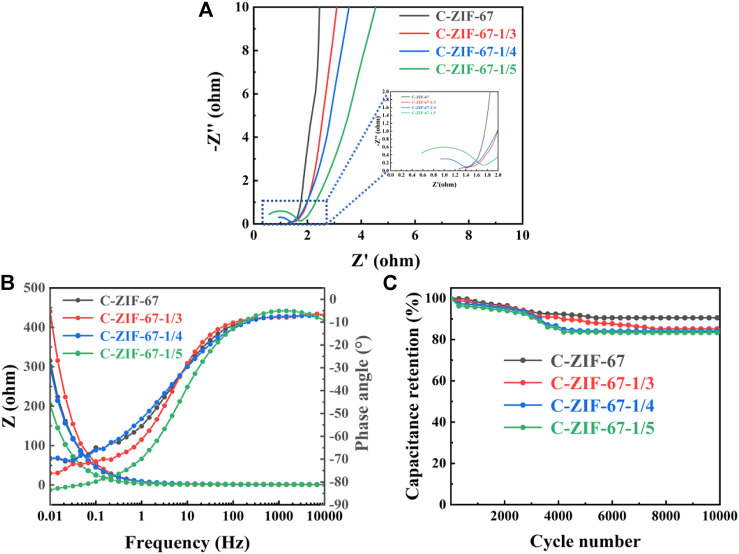
**(A)** AC impedance curve, **(B)** Bode phase angle and electrochemical impedance plots and **(C)** Capacitance retention rate after 10,000 cycles for C-ZIF-67, C-ZIF-67-1/3, C-ZIF-67-1/4 and C-ZIF-67-1/5.

Representative Nyquist plots (from EIS measurements) for the samples are displayed in Figure 7A, in which all samples showed a circular arc-like shape in the high-frequency region. And the C-ZIF-67 sample has the smallest arc radius, which indicates that it has the smallest charge transfer resistance. The straight line shape is shown in the low frequency region, which is caused by the diffusion control of the reactants or products of the electrode reaction. However, the impedance curve deviates from 45° in the low-frequency region, which possibly due to the induced impedance caused by the uneven surface of the electrode. The slope of C-ZIF-67 was the largest, indicating that the material exhibited the best conductivity (Jiao et al., 2022). The C-ZIF-67 sample exhibits excellent low impedance behavior both in the high frequency region and low frequency region. This stems from its special hollow structure, which promotes charge transfer and mass transfer (Yang et al., 2022).

The Bode phase angle plots of C-ZIF-67, C-ZIF-67-1/3, C-ZIF-67-1/4 and C-ZIF-67-1/5 are presented in Figure 7B. The phase angles of C-ZIF-67, C-ZIF-67-1/3, C-ZIF-67-1/4 and C-ZIF-67-1/5 in the high-frequency region were about −70°, −76°, −70° and −81°, respectively. These angles were between −90° (ideal capacitor) and −45° (pseudo-capacitor), which indicated intercalation capacitance in the materials (Krishnamoorthy et al., 2018). Meanwhile, the capacitance retention of C-ZIF-67-1/3, C-ZIF-67-1/4 and C-ZIF-1/5 achieved 87%, 84% and 85%, respectively, and the C-ZIF-67 reached 90%, after 10,000 cycles (Figure 7C). These results illustrate the excellent cycling stability and electrochemical reversibility of the C-ZIF-67 sample in particular. Herein, the relatively high retention rate (90%) of C-ZIF-67 indicates that it has advantages as an energy storage material, possibly because its ratio of microporous and mesoporous structures provides efficient ion transmission and storage, while maintaining a stable capacitance (Yin et al., 2020).

## Conclusion

MOF/pre-hydrolysis solution carbon materials were successfully prepared by hydrothermal hybridization with ZIF-67 (Co-MOF) from a pre-hydrolysis solution and a pre-hydrolysis solution after ultrafiltration concentration. The materials showed a hollow structure with a long, hairy tentacle-like appearance and a specific surface area of 235 m^2^/g. The hollow structure provided sufficient space for the shrinkage and expansion of particles. The low density and large specific surface area of the long hairy tentacle structures provided more active sites and a larger contact area for the electrolyte, which shortened the transmission path of electrons and charges. Doping with a small amount (1 wt%) of Co-MOF released and stored active electrons and free electrons through redox reactions, which greatly improved the conductivity of the carbon materials. The Co-MOF/pre-hydrolysis solution carbon materials exhibited an excellent specific capacitance (400 F/g, 0.5 A/g) and stability (90%, 10,000 cycles). The carbon materials prepared by ultra-filtration showed a good specific surface area and capacitance. Upon increasing the concentration, the sample size gradually became uniform, and the specific surface area decreased accordingly. The specific capacitance decreased from 336 F/g for C-ZIF-67-1/3 to 258 F/g for C-ZIF-67-1/5. The results showed that the electrochemical performance of the carbon materials depended on a synergistic effect between their microporous and mesoporous structures, in which the micropores provided storage space for ion storage, and mesopores provided ion transmission channels. The separation of large and small molecules after ultrafiltration concentration produced uniform pore structures, but it also made the micro- and mesopores unbalanced in energy storage applications, which limited the pore transmission and energy storage of ions.

## Data Availability

The original contributions presented in the study are included in the article/supplementary material, further inquiries can be directed to the corresponding author.
